# Meaningful in-training and end-of-training assessment: The need for implementing a continuous workplace-based formative assessment system in our training programs

**DOI:** 10.12669/pjms.38.5.5921

**Published:** 2022

**Authors:** Laima Alam, Mafaza Alam, Muhammad Najm-ul-Hasan Shafi, Shaista Khan, Zahid Mehmood Khan

**Affiliations:** 1 Laima Alam, FCPS Gastroenterology, MRCP (UK), Bahria Town International Hospital, Rawalpindi, Pakistan; 2 Mafaza Alam Registrar Operative Dentistry and Endodontics, AFID, Rawalpindi, Pakistan; 3 Muhammad Najm ul Hasan Shafi, FCPS Medicine, Islamic International Medical College, Rawalpindi, Pakistan; 4 Shaista Khan, FCPS Radiology, KMU Institute of Medical Sciences, Kohat; 5 Zahid Mehmood Khan, FCPS Operative Dentistry and Endodontics, AFID, Rawalpindi, Pakistan

**Keywords:** Clinical performance, Examinations, Formative assessment, Portfolio, Postgraduate training, Workplace

## Abstract

**Objectives:**

To analyze the systems and tools involved in assessment of skill procurement and demonstrating workplace skills in postgraduate medical training.

**Methods:**

This cross-sectional survey was carried out by enrolling trainee doctors currently working in Medical, Surgical, Dental and Allied specialties of the country by sending a validated and piloted questionnaire through email. Data collection was done from 20^th^ April to 20^th^ May 2021. Data was analysed using SPSS v. 21.0.

**Results:**

A total of 351 completed responses were received from 10 major cities of the country. Multiple aspects of entry-into-training, in-training and end-of-training evaluation showed poor correlation with the required training goals. A comparison of assessment for entry-into-supervised training (FCPS-I) versus independent practice (FCPS-II) showed a dismal situation regarding assessing affective skills like leadership, teamwork, coping with pressure and self-awareness. The concept of maintaining portfolios was completely alien to the trainees and the assessment tools used for demonstrating workplace skills were outdated. The lack of a continuous, periodic and balanced assessment (65%); detailed feedback (61.5%); fair exams (59%); variability in scoring system (58%) and professionalism of the examiners (57.5%) were the most frequently selected perceived flaws in the assessment system by the participants.

**Conclusion:**

There are multiple lacunae regarding competency-based assessment systems in our training programs and a massive scope for improvement. Assessment systems should be implemented as continuous process of learning, self-reflection, feedback and revalidation throughout the training tenure at regular and multiple points.

## INTRODUCTION

Postgraduate assessment for trainee doctors have rationalized from the mere objective of binary outcome (that is pass or fail) to a much wider concept of competency and an insight in to the clinical acumen in real-life scenario.^1^ The Western world is in a continuous process of harmonizing post-graduate training, making it effective, robust and trainee/specialty oriented to enhance learning in competency based programs.^2^ Testing factual knowledge alone has been seen to fail abysmally in assessing skills and behaviour with orthodox examination systems that mainly involve long essay questions, viva voce or performance in a controlled environment by the end of training program.^3^

Our training programs lack objective and continuous systematic assessments for many of the specialties, unfortunately. Even if the trainees are provided with workplace based assessments (WBA), they do not count towards the final postgraduate examinations.^4^,^5^

This study was designed to analyze the systems involved in assessment of skill procurement and growth and the tools used for demonstrating workplace skills during training programs provided by the institutions in Pakistan. Also, the perceived flaws in the assessment systems of the training programs were explored in detail.

## METHODS

This cross sectional survey was carried out by enrolling trainee doctors working in different set-ups of Pakistan through consecutive sampling after acquiring ethical approval from the concerned department (IRB 90/Trg-ABP1K2 dated 20.04.2021). The survey was completed in one month from 20^th^ April to 20^th^ May 2021 by sending a validated and piloted questionnaire through email to trainee doctors currently working in Medical, Surgical, Dental and Allied specialties. Trainees from basic medical sciences, non-trainee doctors and incomplete surveys were all excluded.

The questionnaire was developed by LA and MA after a thorough literature review^4^-^8^ and was reviewed by two medical education experts for content validity. The survey was piloted among 10 post-graduate residents before putting it to test. The questionnaire encompassed questions regarding in-training and end-of-training evaluation systems offered through-out the tenure in our training programswith assessment of skill procurement and growth for entry in to supervised training (FCPS I) versus independent practice (FCPS II). The participants were also surveyed about the various tools used for workplace based assessments (WBA) provided by their training institutes. Also the perceived flaws in the assessment system of the training programswere explored in detail.

The sample size was calculated with margin of error set at 5.5%, confidence level at 95% and an anticipated frequency (response distribution) of 50% using OpenEpi sample size calculator. The questionnaire was sent through email, a reminder was given to the participants after one week of no response and the candidates were dropped who failed to respond after another seven days^6^. Qualitative data was expressed as frequencies and percentages. A value of <0.05 was considered statistically significant. All analysis was done using SPSS V.21.

## RESULTS

**Fig.1:**
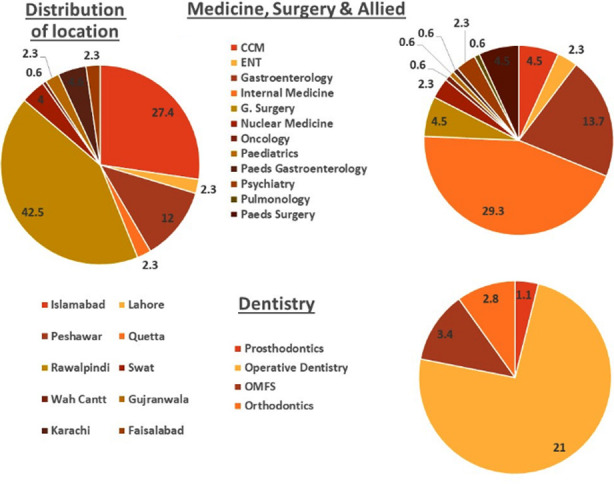
Distribution of specialties and cities of residency.

A total of 351 completed responses were received out of 800 questionnaires sent, making a response rate of 44%. Male participants (76.6%), age range of 25-30 years (68%), working in private setup (39.6%) and those currently in their first fellowship (70%) made majority of the sample (Table-I). The distribution of specialties and region of training are shown in Fig.1.

**Table I:** Demographic variables of the participants.

Variables		Frequency n(%)
Gender	Male	269(76.6)
	Female	82(23.4)
Age (years)	25-30	239(68)
	31-35	94(26.8)
	36-40	10(2.8)
	>40	8(2.3)
Postgraduate degree(s)	Currently in 1^st^ fellowship	245(70)
	Completed 1^st^ fellowship	82(23)
	Currently in 2^nd^ fellowship	14(4)
	Completed 2^nd^ fellowship	10(2.8)
Year of residency	1^st^	76(21.7)
	2^nd^	70(20)
	3^rd^	34(9.7)
	4^th^	24(6.8)
	5^th^	14(4)
	Training completed	133(38)
Set-up	Government	124(35.3)
	Private	139(39.6)
	Army	88(25)

Only 28.5% of the trainees reported the presence of periodic assessments provided by the training institute with only 23% of these assessments counting towards the end-of-training evaluation (FCPS-II) (Table-II). There was a serious dearth of evaluation post compulsory rotations (35%), individual learning plan (IPL) (15%), assessment systems related to training goals (22%), assessment system that helps to identify trainees in need of assistance (22%) and prompting in the form of letters of recommendation/appreciation (22%). A statistically significant number of trainees reported variability in scoring system, professionalism of the examiners, lack of post-assessment feedback and lack of training and revalidation for the assessors during end-of-training assessment.

**Table II:** Assessing in-training and end-of-training evaluation at different institutions.

Survey questions	Yes	No	Don’t know	P value
Entrance exam for supervised training by the institution	188(53.6)	155(44.2)	8(2.3)	<0.001
Periodic assessment provided by the institution	100(28.5)	208(59.3)	38(10.8)	<0.001
Periodic assessment that counts towards the final exam	80(22.8)	245(73)	26(7.4)	<0.001
Provision of clear performance criteria	170(48)	123(35)	58(16.5)	<0.001
Examination that encompasses all the attributes of training program	154(44)	147(42)	50(14.2)	<0.001
Balanced assessment demonstrated at appropriate level throughout training	120(34)	161(46)	70(20)	<0.001
Provision of blue-prints of course curricula	156(44.4)	157(44.7)	38(10.8)	<0.001
Provision of year-wise learning objectives	174(49.6)	155(44)	22(6.3)	<0.001
End-of-rotation assessment	124(35.3)	187(53)	40(11.4)	<0.001
Individual learning plan	52(15)	245(70)	54(15)	<0.001
Provision of assessment system related to training goals	76(21.7)	227(64.6)	48(13.7)	<0.001
Support to progress at your own pace by measuring progress in achieving competencies for the chosen career path	116(33)	167(47.6)	68(19.4)	<0.001
Assessment system that helps to identify trainees in need of assistance	78(22)	217(62)	44(12.5)	<0.001
Prompting for better performance	78(22)	218(62)	56(16)	<0.001
Variability in scoring system during end-of-training assessment	181(51.5)	58(16.5)	112(32)	<0.001
Variability in professionalism of the examiners during end-of-training evaluation	241(68.6)	20(5.7)	90(25.6)	<0.001
Post-assessment feedback during end-of-training evaluation	12(3.4)	277(79)	62(17.7)	<0.001
Laid-back attitude of trainees towards career building	173(49)	88(25)	90(25.6)	<0.001
Laid-back attitude of assessors towards trainees’ performance	143(40.7)	112(32)	96(27.4)	0.02
Training and revalidation for the assessors	94(27)	112(32)	136(39)	0.02

**Fig.2:**
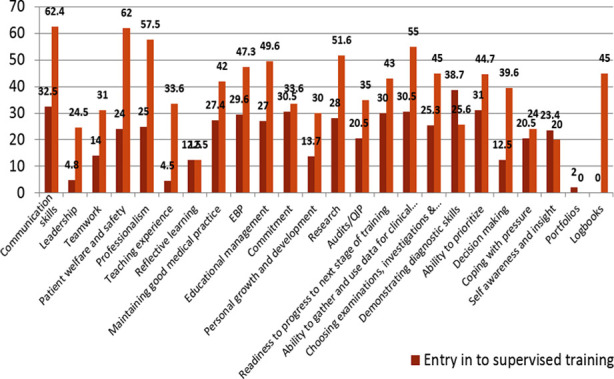
A comparison of assessment of skill procurement and growth for entry in to supervised training (FCPS I) versus independent practice (FCPS II).

**Fig.3:**
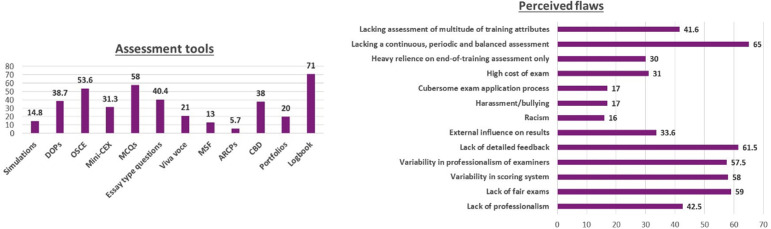
Tools used for demonstrating workplace skills and perceived flaws in the assessment system of the training programs.

A comparison of assessment for entry in to supervised training (FCPS-I) versus independent practice (FCPS-II) in Fig.2 showed a dismal situation regarding assessing affective skills like leadership, teamwork, coping with pressure and self-awareness. The concept of maintaining portfolios was completely alien to the trainees and the assessment tools used for demonstrating workplace skills were, unfortunately, outdated (Fig.3).

The lack of a continuous, periodic and balanced assessment (65%); detailed feedback (61.5%); fair exams (59%); variability in scoring system (58%) and professionalism of the examiners (57.5%) were the most frequently selected perceived flaws in the assessment system of training programs (Fig.3).

DOPS Directly Observed Practical Skills, OSCE, Mini-CEX mini Clinical Examination, MCQs Multiple Choice Questions, MSF Multi-Source Feedback, ARCPs Annual Review of Competence Progression, CBD Case Based Discussion

## DISCUSSION

A continuous, periodic and balanced WBA has notoriously been neglected to be incorporated in to the postgraduate training programs in many Asian countries where the evaluation system relies heavily upon end-of-training summative assessment^5^. Also assessing attitude has always been considered to be of lesser importance than assessing knowledge, cognitive skills and psychomotor skills in our education system. The traditional assessment systems usually underestimate the practicality and the profound effect affective skills have to play in re-humanizing patient-doctor relationship.^7^

It was seen in this study that 45% of the trainees reported non-availability of clear guidelines/blue-prints for their training programs, although the trainee-portal specifically designed by College of Physicians and Surgeons of Pakistan (CPSP) has clear guidelines regarding the competencies and syllabi for all specialties.^8^ This reflects the careless attitude of the majority of our postgraduate trainees who need to inculcate a culture of active engagement in their evaluations and revalidations.

This survey showed that 41% of the trainees believed their clinical supervisors had a “laid back” attitude towards their training needs which emphasizes the need for dedicated mentors who are interested in teaching and identifying trainees that need assistance. One of the methods to accomplish this is to provide the trainees with support to progress at their own pace by measuring progress in achieving competencies for the chosen career path.^9^

Entry-into-training is vigorously controlled and assessed in the West with weighting given to additional post-graduate and under-graduate degrees, awards, teaching experience, publications, presentations/posters/conferences, audits and quality improvement projects and properly compiled portfolios^10^. Also the progression to independent practice is carefully scrutinized with a set of learning outcomes called “capabilities in practice”.^10^ In comparison, our assessment systems lack the basic essence of competency building.

Only 28.5% of the trainees in our study conveyed that WBA are arranged for them periodically by their institutions with assessment tools that were unfortunately outdated. Those trainees who did report periodic assessments revealed that none of these assessment records contributed towards their final exam (FCPS-II), which is a despairing but highly concerning reality. This dismal situation was iterated by a similar study from India that reported evaluation of the trainees only on the day of final examination and none of the workplace based assessment records contributed towards the final assessment.^11^

Portfolios make one of the essential tools to evaluate the progress of a trainee in addition to the fact that they help the trainees to assess themselves by reflection and keep them focused on their learning goals.^12^ Our trainees were alien to the concept of portfolios with no access to reflective learning and have long been entangled in the concept of spoon-feeding and apprenticeship. This cycle of self-pity and holding others responsible for personal failure and lack of goals needs to be terminated with objectivisation of assessment programs.^13^

There is an interesting study by Andreassen P et al addressing the challenges and resistance towards implementing a properly planned assessment program among both trainers and trainees. Subtle tactics to sabotage a periodic workplace based formative assessment like contesting, avoiding and deprioritizing were reported by the authors either because of lack of knowledge or display of hierarchical and authoritative roles by the seniors.^14^ Although this phenomenon was not studied in detail in our study, a significant number of trainees did report a lack of interest on part of their supervisors towards their career goals and requirements. This highlights the role of Educational Supervisors, specifically trained and hired for the sole purpose of evaluating trainees, giving feedback and mitigating the stigma around regular assessments.

Supervisors are required by the CPSP to submit their trainees’ three monthly progress^8^ but unfortunately only 23% of the trainees reported an actual implementation of the supervisor’s crucial role in updating the College regarding their trainees’ progress. The failure rate of FCPS is higher than any of the comparable examinations in the West. Lack of objectivity of assessment systems, personal attributes of the examiners, lack of structured training and no revalidations for the teaching faculty were some of the plausible causes that were thoroughly reviewed by Farooq S.^15^ Similarly, an elaborate report comparing the performance during MRCP exam of UK trained Internal Medicine trainees with those trained outside UK showed a high disparity between the pass-rate (59% versus 32.6%). This emphasizes the need for rigorous standardization of training and assessment programs for the international medical graduates (IMGs).^16^

There were some concerning points raised by the trainees like external influence on results, variability in professionalism of the examiners and scoring system and the lack of fair exams. These are serious allegations and need to be investigated sensitively and meticulously.

Our concern is with the actual implementation and usage of the assessment towards final evaluation to make it a meaningful drive. Without any incentive, neither the residents nor the mentors would exert any effort towards a real change^17^ Career progression should not be considered a burden like summative exams. A culture of self-reflection and self-progression need to be inculcated so that the trainees are involved whole heartedly in their career^18^.There is a need for a shift of focus from hierarchical mentality and apprenticeship towards a teaching culture that promotes competence-based learning and improves clinical abilities.

### Limitations:

The study has its limitations such as the sampling technique and the participants were asked to forward the survey through email for maximum participation. Although the trainees’ grievances were extensively sought in this study, the financial; clerical; time and constraints regarding expertise that need to be endorsed by the higher authorities for running and maintaining the system were not studied.

## CONCLUSION

There are multiple lacunae regarding competency-based assessment systems in our training programs and a massive scope for improvement. Assessment systems should be implemented as continuous process of learning, self-reflection, feedback and revalidation throughout the training tenure at regular and multiple points. Training the trainers as well as the trainees for creating an environment that is safe, using assessment systems that are evenly rewarded and that contribute towards the final assessment while acknowledging the limitations of the system/training facility and the students are the critical steps towards development.

### Author Contribution:

**LA, MA:** Contributed to the idea, questionnaire and data collection.

**LA:** Contributed to the design, statistical analysis and drafting of the manuscript.

**MNHS, SK, ZMK:** Contributed to data collection and critical review.

All authors take equal responsibility for the accuracy and integrity of the work.
